# Molecular and Cellular Mechanisms Involved in Aortic Wall Aneurysm Development

**DOI:** 10.3390/diagnostics13020253

**Published:** 2023-01-10

**Authors:** Iris Bararu Bojan (Bararu), Carmen Elena Pleșoianu, Oana Viola Badulescu, Maria Cristina Vladeanu, Minerva Codruta Badescu, Dan Iliescu, Andrei Bojan, Manuela Ciocoiu

**Affiliations:** 1 Department of Pathophysiology, Morpho-Functional Sciences, Faculty of Medicine, ‘Grigore T. Popa’ University of Medicine and Pharmacy, 16 Unirii Street, 700115 Iași, Romania; 2 Department of Internal Medicine, Faculty of Medicine, ‘Grigore T. Popa’ University of Medicine and Pharmacy, 700115 Iași, Romania; 3 Department of Clinical Cardiology, ‘Prof. Dr. George I.M. Georgescu’ Institute of Cardiovascular Diseases, 700503 Iași, Romania; 4 Department of Surgical Sciences, Faculty of Medicine, ‘Grigore T. Popa’ University of Medicine and Pharmacy, 700115 Iași, Romania

**Keywords:** aortic aneurysms, molecular mechanism

## Abstract

Aortic aneurysms represent a very common pathology that can affect any segment of the aorta. These types of aneurysms can be localized on the thoracic segment or on the abdominal portion, with the latter being more frequent. Though there are similarities between thoracic and abdominal aortic aneurysms, these pathologies are distinct entities. In this article, we undertook a review regarding the different mechanisms that can lead to the development of aortic aneurysm, and we tried to identify the different manners of treatment. For a long time, aortic wall aneurysms may evolve in an asymptomatic manner, but this progressive dilatation of the aneurysm can lead to a potentially fatal complication consisting in aortic rupture. Because there are limited therapies that may delay or prevent the development of acute aortic syndromes, surgical management remains the most common manner of treatment. Even though, surgical management has improved much in the last years, thus becoming less invasive and sophisticated, the morbi-mortality linked to these therapies remains increased. The identification of the cellular and molecular networks triggering the formation of aneurysm would permit the discovery of modern therapeutic targets. Molecular and cellular mechanisms are gaining a bigger importance in the complex pathogenesis of aortic aneurysms. Future studies must be developed to compare the findings seen in human tissue and animal models of aortic aneurysm, so that clinically relevant conclusions about the aortic aneurysm formation and the pharmacological possibility of pathogenic pathways blockage can be drawn.

## 1. Introduction

Aortic aneurysms represent a very common pathology that can affect any segment of the aorta. These types of aneurysms can be localized on the thoracic segment or on the abdominal portion, with the latter being more frequent. Although there are similarities between thoracic and abdominal aortic aneurysms, these pathologies are distinct entities.

In this article, we reviewed the different mechanisms that can lead to the development of aortic aneurysm, and we tried to identify the different manners of treatment.

Aortic aneurysm are pathological conditions that involve focal areas with permanent dilatation of the aortic wall and are most commonly localized in the infra-renal regions and also in the proximal thoracic aortic segments. For a long time, they may evolve in an asymptomatic manner, but this progressive dilatation of the aneurysm can lead to a potentially fatal complication consisting in aortic rupture. Because there are limited therapies that may delay or prevent the development of acute aortic syndromes, surgical management remains the most common manner of treatment. Even though surgical management has improved much in the last years, thus becoming less invasive and sophisticated, the morbi-mortality linked to these therapies remains increased [1,2,3].

Therefore, it is imperative to identify the mechanisms that are linked to aneurysm development in order to be able to improve the medical approach so that in delays or it substitutes surgical treatment. The identification of the subcellular mechanisms and molecular networks triggering the formation of aneurysm would permit the discovery of modern therapeutic targets.

**Figure 1:**
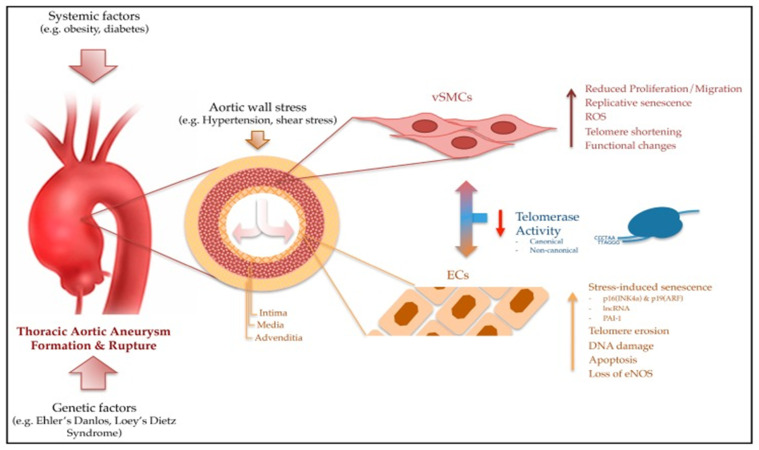
Mechanism involved in the development of aortic wall aneurysms [2].

In order to form an aortic aneurysm, the medial layer of the aortic wall degenerates and becomes thinner, and the elastic lamina deteriorates progressively, thus leading to a diminishment in the tensile strength of the arterial wall (Figure 1).

**Figure 2:**
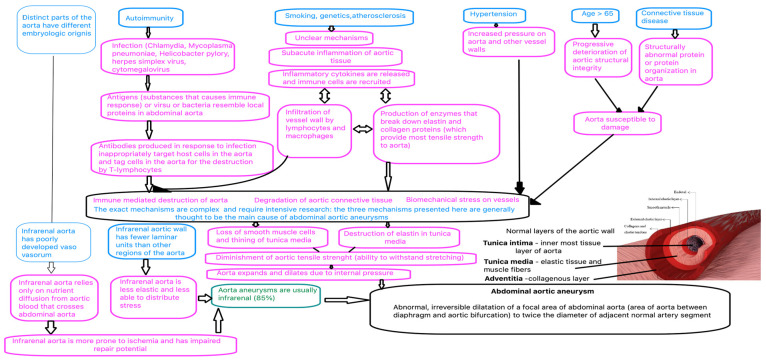
Pathogenesis of AAA—https://calgaryguide.ucalgary.ca/abdominal-aortic-aneurysm-pathogenesis/. Accessed on 14 November 2022.

Abdominal aortic aneurysm (AAA) is linked to the presence of diverse risk factors consisting in dyslipidemia, male gender, older age, hypertension, and smoking. The development and evolution of AAA is an active process that consists of a chronic inflammatory pathology in which hematopoietic cell infiltration, leading to the destruction of the extracellular matrix and vascular structures, can be observed. A relationship between the inflammatory cells and breaks in the elastic lamina associated with reactive oxygen species (ROS) has been observed (Figure 2). This link suggests that the development of AAA is an indolent process that can determine aortic rupture when reaching a stress point [4,5,6].

The incidence of thoracic aneurysm is much lower and their formation may be linked to a heritable pattern as one in five patients diagnosed with thoracic aneurysm has a family history of the same pathology. Even more, it was observed that first-degree relatives had a 10-fold increased risk of developing thoracic aneurysms. Different syndromes have been described to be associated with aneurysm of the thoracic aorta such as Marfan syndrome (MFS), Loeys–Dietz syndrome (LDS), Ehlers–Danlos syndrome (EDS), familial thoracic aortic aneurysms and dissections (TAAD), autosomal dominant polycystic kidney disease (ADPKD), bicuspid aortic valve (BAV), and neurofibromatosis type 1 (NF1).

Thoracic aorta aneurysm is most frequently associated with MFS, but all these syndromes can be used in order to understand the pathogenesis of aortic wall degeneration in aortic aneurysms [7,8,9,10].

## 2. Anomalies of Extracellular Aortic Matrix

The aortic extracellular matrix has high concentrations of elastin and collagen and has an important role in organizing the abdominal aortic wall. In order to develop an abdominal aortic wall aneurysm, the elastin becomes fragmented, and the collagen fibril organization becomes abnormal. These mechanisms are considered to have a major role in aortic dilatation and subsequent rupture. The lysyl hydroxylase (LH1) molecule seems to have an important role in the pathophysiology of aortic wall dilatation. It is encoded by the procollagen-lysine, 2-oxoglutarate 5-dioxygenase 1 (*PLOD1*) gene and it is an enzyme catalyzing the hydroxylation of lysyl residues in Xaa-Lys-Gly. LH1 is responsible for the hydroxylation of residues containing lysyl in collagen-like peptides; this reaction is essential for the stability of the intermolecular cross-links that induce tensile strength in the collagen fibrils and confer mechanical stability [11].

LH is known to have three isoforms (LH1, 2, and 3); it has been shown that LH1 and LH3 may have substrate preferences. LH1 preferably links to type I/III collagen. Mutations of the *PLOD1* gene determine deficiencies of LH1 in humans, the result consisting in the onset of different extracellular matrix disorders such as kyphoscoliotic subtype (subtype VIA) of Ehlers–Danlos syndrome (EDS). These conditions have been associated with life-threatening vascular incidents consisting of aortic dilation and rupture. Research conducted on animal models showed that *Plod1^−/−^* mice had an increased risk of aortic rupture compared with wild-type (WT) mice. A mouse model consisting of angiotensin II (Ang II) infusion was used for preclinical aneurysm research [12,13].

There is increased evidence suggesting that the lesions determined by Ang II in mice can be considered as pseudoaneurysms or dissecting abdominal aortic aneurysms because some relevant differences have been observed in human AAA. The research conducted by Li et al. reported a new defensive function for LH1 against dissecting AAA; the absence of LH1 determines an upregulation of thrombospondin-1 (encoded by *Thbs1*) expression, thus promoting the proinflammatory process, increasing matrix metalloproteinase (MMP) activity, and severe apoptosis of vascular smooth muscle cells in the abdominal aorta, the final result being the dissecting AAA formation. Decreased levels of LH1 lead to structural damage of the aorta in response to Ang II administration. Elastin staining and quantitative analysis showed that low levels of LH1 increased fragmentation of the elastin layer in response to Ang II infusion. Low levels of LH1 have been proven to aggravate gene transcription abnormalities in the abdominal aorta wall. Even more, it was found that a total of 108 genes were expressed in a different manner between *Plod1^−/−^* and wild-type mice after Ang II administration; this mechanism may be responsible for the underlying condition of LH1 deficiency that enhances the risk of developing dissecting AAA [11,14,15].

## 3. Matrix Metalloproteinases

The similarity between AAA and TAA (thoracic aortic aneurysms) consists of a diminishment in elastin, collagen, and glycosaminoglycans compared to the structure of the normal aortic wall. This may be due to an excess of metalloproteinases (MMPs) associated with low inhibitors levels. Existing data have mentioned the presence of 23 distinct MMPs, which can be divided in archetypal, matrilysins, gelatinases, and furin-activated MMPs [16,17,18,19,20]. The normal aortic wall contains endothelial cells, smooth muscle cells, and fibroblasts localized in the adventitia that are responsible for the production of MMPs. In AAA, the level of inflammatory cells increases, thus also leading to an increased production of MMPs.

High levels of MMP-1 (collagenase-1) have been described in AAA. Furthermore, low levels of MMP-1 inhibitors have been found in these patients. Fibroblasts and smooth muscle cells are responsible for MMP-1 synthesis and MMP-3 with plasmin is needed for their activation. The inflammatory cells are only responsible for a minor secretion of MMP-1 [21,22]. Increased levels of MMP-1 and MMP-13 are associated with aortic wall regions that are poor in smooth muscle cells but have increased inflammation of the adventitia. These sites of abnormality can be detected through PET F-FDG uptake and correlate with an increased risk of wall rupture.

MMP-13 is also known as collagenase-3 and has increased levels in AAA that are symptomatic or that are close to rupture. Smooth cell muscles that are found in the aortic wall are the main source of MMP-13 production. A genetic polymorphism (−77A/G) that modifies MMP-13 levels has been proven to be an independent risk factor for aortic wall degeneration in AAA. Higher levels of MMP-13 can be caused by nitric oxide induced synthesis of CD147, the result being a misbehavior of elastases in the aortic wall with degeneration and AAA formation. CD147 can be inhibited through RNA interference or pharmacological blockage of iNOS. Therefore, the pharmacological modulation of these mechanisms can be used to decrease aortic dilatation [23].

MMP-3 is also known as stromelysin-1, and its levels are also increased in the aortic wall of abdominal aneurysms. Epithelial cells and fibroblasts, but also macrophages are responsible for the production of these enzymes in aortic wall aneurysms. An MMP-3 gene promoter region polymorphism known as 5A/6A (5 adenines vs. 6 adenines at −1612) has been described, leading to increased transcriptional activity that determines aortic wall degeneration and abdominal aortic aneurysm formation [24,25].

MMP-12, known as metalloelastase, is formed by macrophages and its implication in aortic wall degeneration remains unclear. Recent studies using the CaCl_2_ model of AAA formation in mice with a genetic inactivation of PI3-kinase delta have shown a significant upregulation of MMP-12 expression, leading to increased AAA formation [19,20,21]. The aneurysmal wall may contain CD68-positive macrophages associated with MMP-12. Genetic inactivation of PI3-kinase delta or treatment with an inhibitor of PI3-kinase delta increases macrophage migration and modulates MMP-12 expression. It is not clear whether MMP-12 is directly linked to aortic wall degeneration, but it seems to increase the action of MMPs responsible for extracellular matrix degradation.

MMP-2, also called gelatinase A, is formed by fibroblasts, smooth muscle cells, and macrophages and is responsible for elastin degradation. Its activity is increased by Ang II and CaCl_2_, and therefore leads to the degeneration of the aortic wall [26,27].

MMP-9, or gelatinase B, results from smooth muscle cells, fibroblasts, and macrophages, and their plasmatic levels and RNA expression are much higher in the AAA wall and in the luminal thrombus. Furthermore, recent research has shown a link between MMP-9 gene expression and the genic expression of inflammatory mechanisms and cholesterol metabolism [28,29,30,31].

MMP-14, or membrane type 1-MMP, results from smooth muscle cells and macrophages present in the aortic wall. It has been shown that MMP-14 can induce direct degradation of the extracellular matrix localized in the adventitia and in the medium tunica. These molecules can influence the elastolytic activity of macrophages [32].

As a conclusion, the existing literature has shown that MMP molecules are important hallmarks for the pathogenesis of aortic wall degeneration, but they are not sufficient to induce by themselves the development of aortic wall aneurysms as they need to be associated with other pathophysiological mechanisms.

## 4. The Role of Inflammatory Cells

The morpho pathological anomalies found when analyzing the aortic wall sample obtained through surgery consist of infiltration with leukocytes, extracellular matrix degradation, and disfunctions of smooth muscle cells. In AAA, the inflammatory process is localized transmurally and consists of macrophages and lymphocytic migration associated with a small increase in mast cells and neutrophils. The aortic media and adventitia are the structures that are mostly affected. Macrophage inflammation involves a signaling pathway relying on focal adhesion kinase (FAK). FAK increases the secretion of MCP-1 (monocyte chemotactic protein-1) and MMP (matrix metalloproteinase)-9 and enhances the chemotaxis that is mediated by MCP-1. It was shown that the use of the pharmacological inhibition of FAK can reduce macrophage levels in the aortic wall and block CaCl_2_-induced AAA progression. Macrophages can be divided into proinflammatory (M1) and anti-inflammatory (M2) ones and aneurysm formation is linked to an increased level of M1 macrophages. *Tnfα^−/−^* macrophages induce increased concentrations of M2 cytokines in contrast to *Il1β^−/−^* macrophages. When *Tnfα^−/−^* macrophages are infused, but not *Il1β^−/−^* macrophages, the formation of aortic wall aneurysms is inhibited. Aside from macrophage phenotype, epigenetic anomalies including microRNAs (miRs) can also influence the activity of macrophages. Nakao and his colleagues proved that miR-33 was a strong regulator of inflammatory cell function involved in aortic aneurysm formation as mice with a genetic deficiency of miR-33 had a diminishment in AAA formation induced by angiotensin II (AngII) infusion or calcium chloride application [2,32,33].

Aside from the accumulation of inflammatory cells, markers of inflammasomes have been found in the aortic aneurysm wall and in plasma. The activation of the NLRP3-caspase-1 inflammasome cascade determines the degradation of contractile proteins of the arterial wall. When the inflammasome pathway is inhibited by either genetic depletion of *Nlrp3* or *caspase-1* in mice or the administration of glyburide, the development of AAA is inhibited [33,34].

Lymphocytes T CD4+ are very frequent in the end stage aneurysmal wall. As they secrete different types of cytokines, they indirectly control the metabolism of the extracellular matrix and influence macrophage recruitment and protein synthesis. CD40–CD40 ligand interaction is an important pathway that signals the communication between antigen-presenting cells, macrophages, and T cells. The genetic deficiency of the CD40 ligand decreases both AngII-induced AAA formation and macrophage and T cell infiltration and reduces the expression of MMPs [35,36,37].

Neutrophils are the first cells to reach the site of inflammation, therefore, they can be linked to aneurysm formation. He and colleagues have shown the importance of FAM3D (Family With Sequence Similarity 3, Member D), which is a novel chemokine involved in AAA pathogenesis. FAM3D had high levels in both human and mouse AAA tissues. FAM3D deficiency or the inhibition of FAM3D-with neutralizing antibody 6D7 reduced the development of elastase or CaPO4-induced AAA in mice. FAM3D exhibits its effects as it is a dual agonist of FPR (formyl peptide receptor)1 and FPR2, thus leading to macrophage-1 antigen-mediated neutrophil recruitment and the development of AAA [38,39,40].

## 5. Anomalies of Lipidic Metabolism

The low-density lipoprotein receptor (LDLR) is accessed by apolipoprotein E (APOE) in order to realize the clearance of lipoprotein particles from the blood and has a crucial role in lipid and lipoprotein metabolism. LDLR gene mutations are present in familial hypercholesterolemia and type III hyperlipidemia. These conditions significantly increase the susceptibility to develop severe atherosclerosis at a young age [26]. Mutations in the *LDLR* can be located on chromosome 19p13.2, and mutations of the low-density lipoprotein receptor-related protein 1 (LRP1) are located on chromosome 12q13.3; these anomalies induce a genetic susceptibility to develop AAA [27]. Furthermore, several *APOE* polymorphisms modulate the risk to develop atherosclerosis and AAA. The role of APOE 2, 3, and 4 alleles in patients with AAAs has been evaluated and it was proven that the E3/E4 genotype induced a much lower AAA expansion rate than the E3/E3 genotype [41,42].

Normal HDL has been proven to regulate the efflux of cholesterol molecules from tissues and can also modulate inflammation and oxidative stress; therefore, it can be considered as a potent anti-inflammatory and antioxidant molecule. It has, in its constitution, apolipoprotein A-1 (apo A-1), which is a major protein component that shows an anti-inflammatory effect on monocytes as it inhibits the activation of the CD11b molecule. It has been shown that in patients with ascending aortic dilatation and bicuspidia, the levels of LDL cholesterol are lower compared to the patients with no aortic dilatation. The monocytes that are found in the blood stream produce cytokines and molecules that interact with platelets and endothelial cells, thus inducing a protease mediated destruction of the extracellular matrix, with medial apoptosis and smooth muscle cell differentiation; they are also responsible for the increased oxidative stress associated with neovascularization and clotting formation.

As a result of these findings, a new predictor of cardiovascular events entitled monocyte count-to-HDL-cholesterol ratio (MHR) has been established. MHR has been shown to be associated with slow coronary flow, ectasia of the coronary arteries, stent thrombosis, and modified aortic elastic characteristics in hypertensive patients. It is also associated with the size of aneurysm in patients with abdominal aortic aneurysm [43].

The antioxidant effect of HDL is mainly mediated through the inhibition of the LDL oxidation associated with a reduction in the cellular uptake by the phagocytic system of monocyte-macrophages. The antioxidant activity of LDL is multifactorial. HDL seems to exhibit chelation properties as it has proteins such as ceruloplasmin on its surface. The peroxidative lipidic products are a result of oxidized LDL and have been demonstrated to be cytotoxic and to promote atherosclerosis.

HDL molecules have been proven, through in vitro studies, to accept hydroperoxides located in oxidized membranes, thus potentially providing a pathway for excretion or detoxification. Therefore, the ratio between the number of monocytes and HDL can contribute to the effect of oxidative stress in aortic dilatation.

It was also proven that uric acid can be an independent predictor of aortic dilatation. Uric acid is the final product resulting through oxidation during purine catabolism in humans and is known to be a strong predictor of cardiovascular risk and adverse outcome [44]. The research conducted by Tang et al. proved that uric acid levels are linked to aortic root dilatation in hypertensive patients [44].

## 6. The Renin Angiotensin Aldosterone System (RAAS)

The RAA system is linked to aortic wall aneurysm development through Ang II mediated mechanisms. The induction of AAA has been obtained by angiotensin II infusion in apo E and LDL receptor knockout mice, therefore documenting a causal relationship between AAA formation and RAAS. Furthermore, the inhibition of angiotensin II converting enzyme through medication limited AAA formation in the elastase model [45].

Angiotensin II is therefore necessary for aneurysm induction, but not sufficient as other risk factors should be associated in order to modify the aortic wall such as elevated cholesterol, inflammation, hypertension etc. Ang II can be formed through different pathways, the most efficient being the angiotensin converting enzyme formation mechanism. In addition to this pathway, Ang II can be formed by the intervention of chymase expressed in mastocyte cells, leading to a local generation of Ang II. The importance of the chymase pathway remains to be evaluated as it has an additional role in the activation of MMPs and apoptosis [46].

The effects of angiotensin II on the cellular components of the aorta have been thoroughly evaluated and consist of many cellular mechanisms including the production of reactive oxygen species, the induction and activation of MMPs, and infiltration of the aortic wall with inflammatory cells [47]. The clinical result regarding the use of angiotensin converting inhibitors is rather contradictory, as clinical trials did not prove a benefit of this medication on the AAA growth rate [48]. The reason for this discrepancy remains unknown and is probably due to some deficiencies in the available animal models, the effects on aneurysm initiation and progression, or due to the endpoint of the aortic diameter.

The mineralocorticoid receptor seems to play a role in AAA formation as the administration of aldosterone in mice with normal lipidic levels was proven to induce AAA. This effect was age dependent with a more evident phenotype in older animals.

## 7. Endothelial Injury Markers

Some studies suggest that endothelial cell injuries can represent a basic milestone in the process of aneurysm formation. The loss of the integrity of the endothelial layer is able to trigger the infiltration of immune cells, enhancing intraluminal thrombus formation, and influencing the proliferation and migration of smooth muscle cell. The endothelial cells that are injured liberate a variety of soluble particles that can be considered as markers of endothelial damage. These endothelial particles that can be found in the blood stream can be quantified and may be considered as candidates for clinical testing. One of these markers of endothelial damage is a soluble serum thrombomodulin (sTM), which is an endothelial bound protein that has increased levels in pathologies associated with vascular risk such as peripheral and coronary atherosclerosis, diabetes mellitus, rheumatoid arthritis, and systemic lupus erythematosus [48,49].

TM is considered to be one of the most popular indicators of endothelial injury; it is located on the surface of the vascular endothelium and has an anticoagulant effect. TM links to thrombin, forming a 1:1 thrombin–thrombomodulin complex with an inhibitory effect on fibrin formation and platelet activation and protein S activation. TM has both a transmembrane form as well as a soluble plasmatic form (sTM), which is probably produced by the cleavage of the transmembrane glycoprotein. It was demonstrated that endothelial cells release sTM as a result of cell membrane injury induced by the action of neutrophil derived proteases and oxygen radicals. High levels of sTM were observed in clinical conditions that are associated with vascular abnormalities such as atheromatous arterial disease, diabetes mellitus, and different types of active vasculitis. STM is not characterized by a circadian rhythm and is not influenced by age, nor is it influenced after exercise or during an acute response to a variety of biological stimulations. This is why plasma TM can be considered as a specific marker for endothelial lesions and not a molecule indicating endothelial activation [50,51].

Recent research has shown that sTM had increased levels in patients with aortic aneurysm that qualified for EVAR (endovascular aneurysm repair). This result can be due to the fact that EVAR is carried out in elderly patients that have multiple coexisting pathologies, which may act as supplementary factors that disrupt the integrity of the endothelial layer and upregulate the levels of sTM. Significantly increased levels of sTM were noticed 48–96 h after EVAR, but only in patients who had undergone surgery. The sTM levels had a slight tendency to be more increased in EVAR patients compared to patients who underwent open surgery. The surgical procedure can be associated with ischemia-reperfusion (I/R), which is responsible for increased levels of reactive oxygen species (ROS) and can induce systemic inflammation. Endothelial injury results from both ROS and inflammatory molecules. Research conducted by Barry et al. proved that venous blood contains increased concentrations of ICAM-1 when taken during the reperfusion period. The blood taken from AAA patients after reperfusion through surgical repair contains high amounts of integrins. The soluble form of ICAM-1 is extremely increased in AAA patients with shock and in non-survivors after the rupture of AAA [48,52].

The highest levels of VCAM-1 were found in the first 2 days after surgical repair of AAA as the research carried out by Kokot et al. indicates. These results imply that sTM, and VCAM-1 may develop into useful markers in the assessment of early endothelial injury in AAA patients after surgery [53,54].

## 8. Genetic Insights Regarding Aneurysm Development

The normal structure of the elastin contractile unit is mandatory for an efficient aortic function. The signaling mechanism is also very important for normal aortic functionality. If structural and functional anomalies appear, the aorta becomes vulnerable to dilatation and rupture. There are new data suggesting that modified cell-matrix links affecting the contractile elastic unit can lead to the formation of aortic aneurysm. These anomalies can be induced by genetic mutations of the genes responsible for the elastin-contractile unit and for the proteins that are present in the signaling pathways.

Several mutations that can be linked to aortic aneurysm development have been described.

*SM α-actin gene (ACTA2)* is responsible for encoding the smooth muscle cells specific α-actin isoform. The mutations of this gene are present in familial cases of thoracic aortic aneurysm dissections, as associated with medial degeneration and disarray of the medium tunica containing smooth muscle cells. ACTA 2 mutations lead to impaired actin filament assembly, which induced anomalies in the movement of actin filaments and modified the smooth muscle cells contractions.

*Myosin heavy chain 11 gene (MYH 11)* is responsible for encoding the smooth muscle cell specific myosin heavy chain 11, which is present in the thick filaments of the contractile unit. Mutations affecting MYH11 inhibit the polymerization of myosin from polymerizing into thick filaments, thus leading to anomalies in force generation. This mutation also reduces the expression of contractile proteins and induces dedifferentiation of the smooth muscle cells, therefore compromising the response against stress or injury and increasing the risk of intramural damage [55,56,57].

*Myosin light chain kinase gene (MYLK)* is responsible for encoding a molecule that can phosphorylate and activate the myosin regulatory light chain. This process initiates smooth muscle cell contraction as it enhances the ATPase activity of actin-dependent myosin II. When mutations appear, the contraction is compromised and medial degeneration of the aortic wall can be observed.

*Type I cGMP-dependent protein kinase (PKG-1)* is responsible for the activation of the light chain of myosin phosphatase, which will finally lead to smooth muscle cell relaxation. When the function of the gene is enhanced, severe aortic dissection of the descending thoracic aorta can appear [58,59].

*Filamin A gene (FLNA)* is a large protein that binds to actin and links the cytoskeleton to transmembrane integrin molecules, and as a result, maintains cell shape and contraction. When hypo functional, it increases the risk of thoracic aortic aneurysm formation and leads to an Ehlers–Danlos like phenotype.

*Fibrillin 1 gene (FBN1)* is responsible for encoding fibrillin-1, which is a microfibril protein that links the elastic fibers to the dense plaques present in the plasmalemas of the smooth muscle cells. This has a major role in the stability of the elastic tissue. Its mutations are linked to the development of Marfan syndrome, which is frequently associated with aortic wall dilatation. The mutations disorganize the elastin structure and leave fibrilin susceptible to proteolysis-mediated degradation. Furthermore, FBN1 mutations diminish the smooth muscle cell contraction, induce extracellular matrix remodeling, and increase the levels of matrix metalloproteinases, causing elastic fiber destruction. As a final result, the aorta modifies its elastic properties and dilates progressively [60,61].

## 9. Novel Mechanisms Associated with Aortic Aneurysms

### 9.1. Epigenetic Mechanisms Linked to Aortopathy

Aside from the genetic anomalies that lead to aortic dilatation, the epigenetic mechanisms may become important hallmarks in the embryonic development of the heart. When dysregulations of the epigenetic mechanisms appear, different pathologies can emerge. Aberrant epigenetic anomalies have been associated with aberrant gene expressions, causing hemodynamic impairment in the aorta, thus leading to dilated aortopathy. The epigenetic mechanisms induce DNA methylation and modifications of the histones. The methylation of DNA usually affects CpG dinucleotides that are grouped in CpG islands; this mechanism will determine the stable silencing of gene expression [62,63]. Recent research has shown that hypomethylation and hypermethylation of the *ACTA2* and *GATA4* genes are epigenetic-induced mechanisms involved in aortic wall impairment [64]. Another pattern of hypomethylation in genes related to the epithelial-to-mesenchymal transition process was proven to be important in inducing aortic wall remediation. These results underline the importance of epigenetic alterations in promoting the development of aortic aneurysm, but it is not well established until now whether the discordant methylation process is the cause or the consequence of the aortopathy.

The other epigenetic anomaly consists of histone modification, which seems to play a crucial role. Modification of the histone H3 marker near the promoter of the *SMAD2* gene may be observed in patients with thoracic aortic aneurysm; this is considered to be an epigenetic mechanism linked to SMAD2 overexpression [65]. The effects of this modification induce a malfunction of cellular related tissue repair processes associated with pathological extracellular matrix remodeling, disrupted proliferation, and migration caused by the dysregulation of the TGF-β/SMAD pathway, which is very important in vascular remodeling [65].

### 9.2. Importance of MicroRNAs and Other Regulatory RNAs

It is important to evaluate the role of the post-transcriptional mechanisms of regulation that involve non-coding RNAs (ncRNAs) in inducing aortic wall dilatation. Of particular interest are microRNAs (miRNAs) and long non-coding RNAs (lncRNAs).

MiRNAs are 22–25 nucleotide ncRNAs that are responsible for a negative regulation of the synthesis of proteins as they inhibit protein translation or promote the cleavage of mRNAs. To date, studies have proven that an atypical expression of miRNAs is present in distinct cases of aortopathies, and this aberrant expression may be caused by the genic polymorphisms that encode for these miRNAs [66].

The research conducted by Yanagawa et al. reported the presence of 34 out of 1583 miRNAs that can have a different expression, thus underlying the downregulation of miR-141 [67]. Recent studies have suggested miR-3688 and miR-424 as new molecular features that target crucial components of the Hippo and TGF-β pathways; the latter is characteristically dysregulated in aortopathies [67].

Another study also showed that the regulation of specific miRNAs in aortic tissue such as miR-133a and miR-143 can lead to the development of aneurysms in patients with a bicuspid aortic valve due to their effects on MMP/TIMP homeostasis [68].

As a result of all this novel research, miRNAs have arisen to become potential biomarkers for the prediction and prognosis of different aortopathies [62,66,67,68,69].

## 10. Conclusions

In this review, we provide a comprehensive assessment of the molecular and cellular mechanisms of aortic wall degeneration leading to the development of aortic aneurysms. The influence of inherited genetic anomalies on aortic aneurysm development and aneurysm rupture and the multiple environmental risk factors associated with AAA have been repeatedly underlined, but the molecular and cellular mechanisms are gaining a larger importance in the complex pathogenesis of aortic aneurysms. Future studies must be developed in order to compare the findings seen in human tissue and animal models of aortic aneurysm, so that clinically relevant conclusions about the aortic aneurysm formation and the pharmacological possibility of pathogenic pathways blockage can be drawn.
